# Using computer-based habit versus chess-based cognitive remediation training as add-on therapy to modify the imbalance between habitual behavior and cognitive control in tobacco use disorder: protocol of a randomized controlled, fMRI study

**DOI:** 10.1186/s40359-023-01055-z

**Published:** 2023-01-25

**Authors:** Damian Karl, Alfred Wieland, Yury Shevchenko, Nadja Grundinger, Noah Machunze, Sarah Gerhardt, Herta Flor, Sabine Vollstädt-Klein

**Affiliations:** 1 Department of Addictive Behaviour and Addiction Medicine, Medical Faculty Mannheim, Central Institute of Mental Health, University of Heidelberg, 21 20, PO Box 12, 68072 Mannheim, Germany; 2 Institute of Cognitive and Clinical Neuroscience, Medical Faculty Mannheim, Central Institute of Mental Health, University of Heidelberg, Mannheim, Germany; 3 Research Methods, Assessment, and iScience, Department of Psychology, University of Konstanz, Constance, Germany; 4 Mannheim Center for Translational Neurosciences (MCTN), Medical Faculty Mannheim, Heidelberg University, Mannheim, Germany

**Keywords:** Addiction, Smoking cessation, Habitual behavior, Cognitive training, Implicit priming, Pavlovian instrumental transfer, Habits, Impulse control

## Abstract

**Background:**

Although the vast majority of smokers are aware of the enormous preventable health hazards caused by smoking, only a small percentage of smokers manage to remain abstinent in the long term. One possible explanation for this discrepancy lies in the inflexibility of addictive behavior and associated disadvantageous decision‐making. According to a dual‐process theory of decision‐making, two distinct decision systems can be identified. One slow deliberate system based on desirable expectations of outcome value described as goal‐directed behavior and a fast reflexive system based on habitual instrumental behavior and driven by reinforcement experienced in the past. In the course of addiction development, an imbalance occurs between habitual behavior and goal-directed. The present study aims to investigate the modifiability of the balance between habitual and goal-directed behavior at the neurobiological and behavioral level in smokers using two different novel add-on therapies. We hypothesize that both interventions change the balance between goal-directed and habitual behavior, but by different mechanisms. Whereas a cognitive remediation treatment should directly improve cognitive control, in contrast an implicit priming task should affect the early processing and the emotional valence of smoking and smoking cues.

**Methods:**

We will conduct a randomized controlled study in treatment-seeking individuals with tobacco use disorder applying either chess-based cognitive remediation training (N = 30) or implicit computer-based habit-modifying training (N = 30) as add on therapy compared to the standard smoking cessation group therapy (N = 30) only. We will address neurobiological and neuropsychological correlates associated with craving, reward devaluation, cue reactivity and attentional bias. In addition, various effects of treatment and prediction of treatment outcome will be examined using behavioral and neural measures.

**Discussion:**

The present study will apply different examination methods such as functional magnetic resonance imaging, neuropsychological tests, and self-report before and after the interventions. This allows the identification of intervention-specific mechanisms and therefore potential neurobiology-based specific treatment targets for individuals with Tobacco Use Disorder.

*Trial registration*: Registered at clinicaltrials.gov/ct2/show/NCT03764969 (05 December 2018).

**Supplementary Information:**

The online version contains supplementary material available at 10.1186/s40359-023-01055-z.

## Background

Tobacco use disorder (TUD) causes over 5 million deaths per year globally, over twice as many deaths as due to alcohol and illicit drug use combined [1]. Therefore, the World Health Organization has gone as far as to describe the current situation as a “tobacco epidemic” and considers it one of the greatest threats to public health the world has ever faced [2]. The vast majority of smokers are aware of the serious consequences smoking has on their health and, when asked, state that they would like to quit. Therefore, it seems all the more remarkable that only 2–5% of smokers who try to quit are still abstinent after one year [3]. This discrepancy could reflect the inflexible and compulsive nature of addictive behaviour and highlights the central role of goal-directed versus habitual control in addiction [4].

Whereas goal-oriented behaviour is based on decisions that are made in view of expectations about the outcome, habitual behaviour is automatic. It is learned through instrumental reinforcement and carried out even when the associated outcome has an aversive value [5]. Dual-process models of addiction assume that an imbalance between goal-directed and habitual behaviors, which have also been associated with reflective and impulsive behaviors, explains the maintenance of drug-seeking behavior [6]. Addictive behavior can be viewed as the endpoint of transitions from initially hedonic, goal-directed substance use to habitual and ultimately compulsive consumption [4].

On a neural level, goal-directed behavior has been associated with prefrontal cognitive control mechanisms, whereas habitual behavior seems to be mainly controlled by shifts from mesolimbic to dorsal striatal pathways [4, 7]. Thus, the imbalance between goal-directed and habitual behavior in addiction is associated with a dysfunction of fronto-striatal circuits [8, 9]. Frontal control mechanisms involve, among other brain regions, the dorsolateral prefrontal cortex (DLPFC) and the inferior frontal gyrus (IFG) [10]. While goal-directed behaviour depends on a cortico-striatal network, the rigid stimulus–response habit system recruits a striato-nigro-striatal dopamine-dependent loop circuit. The shift of these two networks should not be considered in isolation but instead be integrated into a broader striatal dynamics model based on a shift from ventral to dorsal striatum [11]. In fact, there is evidence from human studies on alcohol of a shift from ventral to dorsal striatal responses, involving a change from hedonic to compulsive drug use [12]. Further, Pavlovian-to-instrumental transfer (PIT) paradigms have shown that originally acquired appetitive Pavlovian associations can be transferred to instrumental responding and can override unfavorable instrumental responses and, vice versa, negative Pavlovian associations can induce unfavorable behavioral inhibition [13]. In the case of substance use disorder (SUD), it has been shown that PIT effects are enhanced in individuals with alcohol use disorder and that the PIT effect was associated with increased striatal activation, which was predictive of the amount of alcohol intake and relapse [14]. To what extent these psychobiological changes also play a role in tobacco use disorder is controversial [15]. Further, it is still unclear whether subgroups of individuals with TUD with different motivational foci might be identifiable—ultimately resulting in individually different treatment needs.

On the one hand, several cognitive and behavioral treatment approaches have been formulated to enhance cognitive functioning and to reduce habitual behavior in substance use disorder. Cognitive remediation treatment (CRT) has been designed to improve cognitive skills relating to executive functioning, such as inhibition, decision making, working memory, cognitive flexibility, and attention [16]. When applied to addictive behaviors, its focus is on enhanced control over craving and impulsive actions. CRT has been successfully used to treat substance abuse [17]. Positive effects were found for attention, working and episodic memory and craving. One version of CRT is chess-based CRT. In the present study, a cognitive intervention based on playing chess will be used. There is good evidence that cognitive control and inhibitory capacity can be enhanced by chess training [18]. Chess has the potential to exercise and strengthen executive functions since a player must continually assess an array of potential consequences resulting from any given move [19]. Typical cognitive challenges associated with substance use disorders, such as impaired problem-solving skills, mental flexibility and working memory, could be re-trained by chess [20], as the game demands continual problem solving, awareness of changing circumstances, the capacity to respond to unexpected countermoves by an opponent, and the ability to plan multiple moves ahead. In addition, chess trains the frontal brain regions that are impaired in substance use disorders. Functional magnetic resonance imaging (fMRI) studies have revealed that the DLPFC, a region relevant to addiction [10], but also pre-motor, parietal, temporal and occipital cortices are activated when players are actively engaged in contemplating the next move, remembering positions of chess pieces on a board, comparing them to previous arrangements, or solving complex chess problems [21–23]. The beneficial effect of chess training on cognition and its therapeutic success was investigated in individuals with cocaine use disorder [24]. Here, so-called ‘motivational chess’ was employed as an add-on therapy, a combination of motivational interviewing [25] and classical chess training. An improvement in attentional and executive functioning was observed. Chess training significantly improved working memory compared to a control group receiving occupational therapy. Consequently, chess-based CRT is suggested to improve inhibitory control and executive functions and should decrease impulsivity. This, in turn, should positively affect the course of therapy and outcome measures (e.g., relapse). We will therefore apply a chess-based CRT in a group-setting where we strengthen cognitive, coal-directed functioning.

On the other hand, cognitive bias modification has been employed to modify habits [26]. SUD Patients were either trained to avoid drug-related stimuli by a motor response [27] or had to re-locate their attention away from drug-related materials [28]. However, the effects of these interventions are small and often do not generalize [29]. The reason for the small effects could be that the stimulus-reaction associations are often based on implicit emotional conditioning processes while the interventions focus on motor behavior or attentional processes. In addition, there is no training of alternative reinforcing stimuli, which seem to be undervalued in states of addiction [30]. In eating disorders, similar dissociations between habit-based and goal-directed behaviors have been observed [31]. There, the method of implicit priming has been used to directly alter the emotional valence and arousal of food-related stimuli [32]. This procedure involves the short subliminal presentation of negative pictures such as dead bodies as primes before the unwanted target item (e.g., a drug or high-calorie food) and of positive pictures such as beach scenes before a wanted item (e.g., non-drug reinforcer or low-calorie food). It could been shown that this priming procedure significantly altered the valence of the food and this effect generalized to a later time point [32]. We will therefore implement a directly habit-oriented intervention, an implicit computer-based habit-modifying training (ICHT), where we will use implicit training to associate smoking cues with subliminally presented negative primes. Additionally we will employ a training of alternative positive activities by combining positive primes with various types of potentially positive activities [33]. Contexts (such as a group of friends) will be paired with the drug cues, because contexts greatly add to the efficacy of cues to elicit drug-related responses [34].

## Methods

### Objectives

This study aims to assess the imbalance between goal-directed and habitual behaviour in treatment-seeking tobacco smokers, while examining the differential modification of its neural basis using two training interventions. The first intervention is a chess-based CRT, also known as cognitive enhancement therapy, focusing on improving inhibitory control and executive functions. The second intervention, a computer-based habit-modifying training focusing on implicit drug seeking (implicit computer-based habit-modifying training, ICHT) uses a conditioning approach through implicit priming and contextual modulation. Indicators of the imbalance will be examined with respect to reward devaluation, cue reactivity and a PIT paradigm.

We hypothesize that both interventions change the balance between goal-directed and habitual behaviour but by different mechanisms. Whereas chess-based CRT should directly improve cognitive control ICHT, in contrast, should affect the early processing and the emotional valence of smoking and smoking cues.

### Setting

To investigate change in the hypothetical imbalance between targeted and habitual behaviour, treatment-seeking individuals with tobacco use (smoking) disorder (TUD) according to fifth version of the Diagnostic and Statistical Manual of Mental Disorders (DSM-5; [35]) will be randomized to three intervention groups. Assessment will take place before (baseline) and after (second investigation day) a six-week intervention period. A third, follow-up assessment will be conducted three months after. All assessments will be carried out at the Central Institute of Mental Health, Mannheim, Germany.

### Study population and design

The sample will comprise 90 right-handed, treatment-seeking smokers (males and females) between 18 and 65 years of age, who will attend a 6-week program for smoking cessation. Recruitment will take place using public advertisement, newspaper articles or web-based public calls for participation. Eligibility criteria include at least four of eleven TUD criteria according to DSM-5, sufficient ability to communicate with investigators and answer oral and written questions and ability to provide written fully informed consent. Individuals will be excluded, if they meet any of the following criteria: severe internal, neurological, and/or psychiatric comorbidities, axis I mental disorders other than TUD according to the International Classification of Diseases (ICD-10) and DSM 5 (except for mild depression, adaptation disorder and specific phobias) in the last 12 months, history of brain injury, severe physical diseases, common exclusion criteria for MRI (e.g., metal, claustrophobia), positive drug screening (opioids, benzodiazepines, barbiturates, cocaine, amphetamines), psychotropic medication within the last 14 days and pregnancy.

**Fig. 1:**
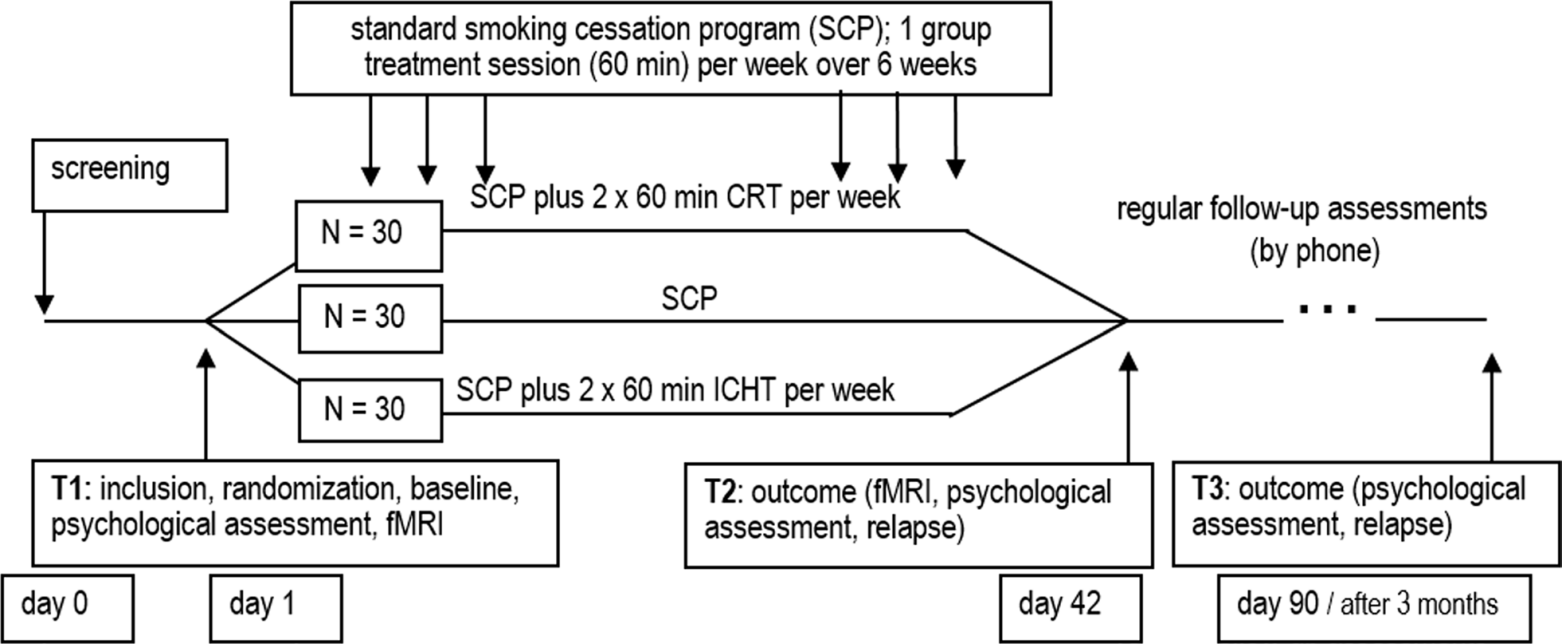
Study schedule and measurement time points. SCP: Standard smoking cessation program; CRT: cognitive remediation treatment; ICHT: implicit computer-based habit-modifying training

90 participants will be randomized into three groups (3 × 30 smokers): Standard smoking cessation program (SCP), standard SCP plus CRT and standard SCP plus ICHT. In addition to the screening for suitability to participate in the study, three investigation days per subject are planned. Participants will be assessed before SCP (Baseline, T1), after a 6-week smoking SCP (T2), and in a detailed follow-up after three months (T3). The comparison will be limited to SCP rather than an additional placebo control group, since the study aims to analyse treatment-related mechanisms rather than general efficacy. See Fig. 1 for more details.

### Sample size

Sample size estimation was conducted using the software package G*Power http://www.gpower.hhu.de), focussing on neurobiological correlates of changes in goal-directed and habitual smoking behaviour following the interventions. Expecting a minimum effect size of f = 0.2 (repeated measures design with within- and between subject factors and interactions), 28 participants per group (SCP, SCP + CRT, SCP + ICHT) will be needed to obtain at least 90% power for our main analyses at 5% alphas level. To counteract possible dropouts, 30 participants per group will be included.

### Interventions

*Standard smoking cessation program (SCP):* Each subject will receive standard SCP as group treatment once a week (1 h) over six weeks. This group therapy is based on behavioral therapy and a psycho-educational approach (for more details, see [36]), and will be carried out by a qualified therapist.

**Fig. 2:**
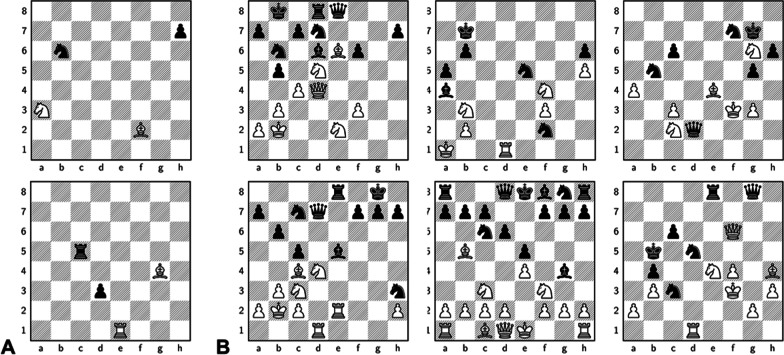
Examples of chess-based CRT (Club de Ajedrez Magic de Extremadura, Mérida, Badajoz, Spain). **A** Short-term memory; memorizing a chess position which is displayed for two minutes; then completing a distractor task: Count backwards in pairs, from 20 to zero; before reassembling the memorized position on an empty demo-board; **B** Focal attention; comparing six chess position with each other and finding similarities and differences

*Cognitive remediation treatment (CRT):* As cognitive remediation treatment we will employ a chess-based battery of tasks two times per week (60 min duration per session) over six weeks as a group treatment in the outpatient clinic of the Central Institute of Mental Health. This training uses a validated battery [37] to strengthen cognitive functioning in specific domains such as short-term memory (see Fig. 2), selective and focal attention, pattern recognition, visuospatial abilities, planification skills, and inhibition. In addition, metacognitive abilities will be trained by explicit teaching strategies (e.g., thinking aloud, giving educational background, discussion underlying cognitive processes) in order to enhance awareness of these aspects (for more details, see [38]). Training of the staff and supervision will be conducted in cooperation with the psychologist Juan Antonio Montero, Club de Ajedrez Magic de Extremadura, Mérida, Badajoz, Spain.

**Fig. 3:**
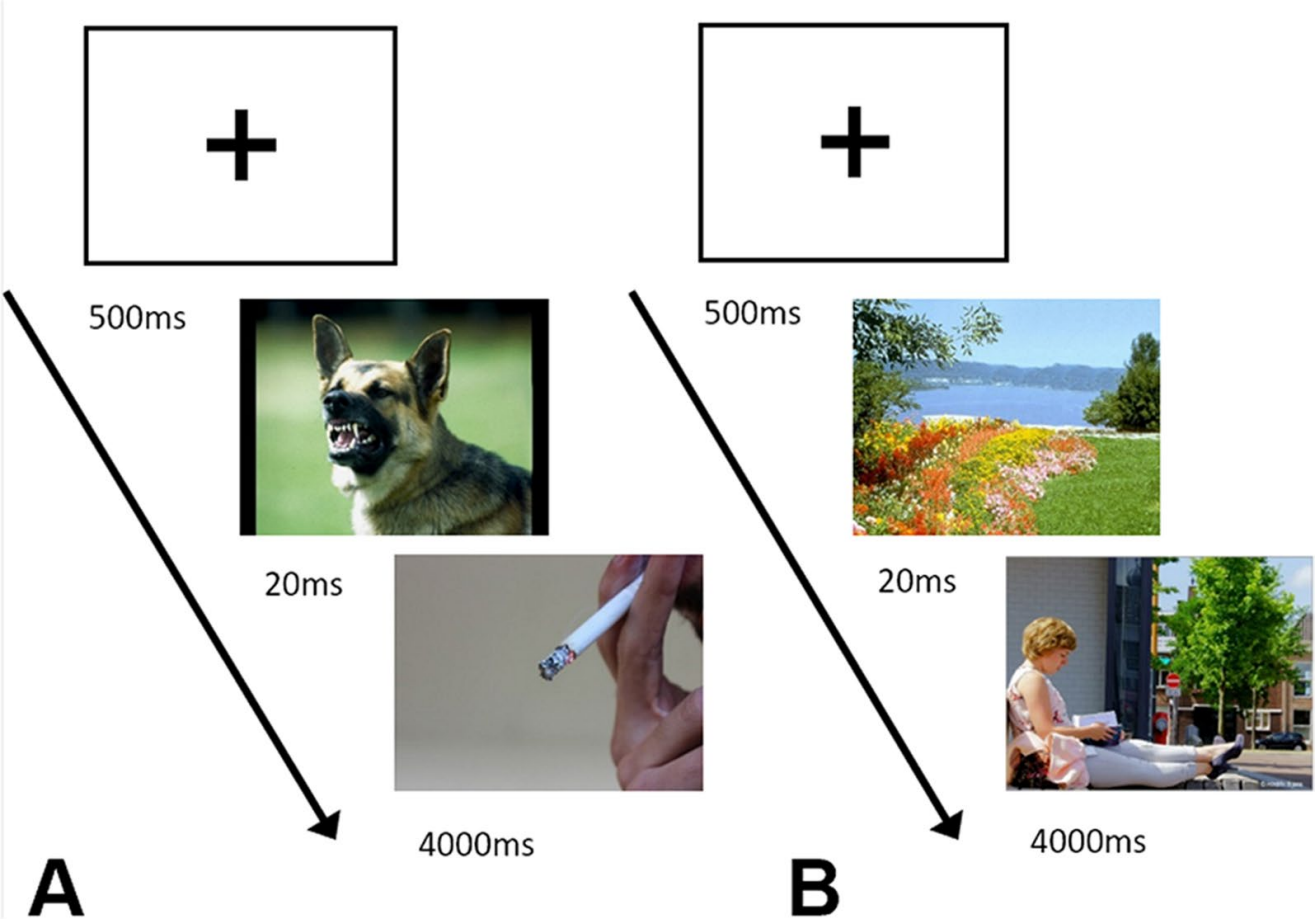
implicit computer-based habit-modifying training (ICHT). **A** Example for subliminal negative valenced prime before an individually chosen smoking cue/context. **B** Example for subliminal positive valenced prime before a picture of an individually reinforcing event

*Implicit computer-based habit-modifying training (ICHT)* Participants will perform a two-part training. In the first part, a subliminal presentation (20 ms) of negatively valenced primes before smoking-cues in context (see Fig. 3) is shown. In the second part, a subliminal presentation of positively valenced primes before potentially reinforcing events, which are individually chosen from the “Pleasant Event Schedule” [33] and complemented by additional contemporary pleasant events. Previous studies observed neural alterations and an effect of implicit priming on food cues in adults with BMI ≥ 25 kg/m^2^ [39] which could lead to healthier eating habits due to alterations in high-caloric food preferences [32]. Through this implicit priming, while further adding an individual context, we expect a decrease in compulsiveness in smoking behavior. To maintain attention to the task, participants are asked to respond to black and white images (by pressing a button) as quickly as possible during the training session. After each training session, participants are presented with six images of smoking cues and six images of reinforcing events. Half of the images are from the session, and the other half are new images. For each of the images, participants have to indicate whether they saw that image during the session. Participants also rate the valence and arousal of the images using the Self-assessment Manikin [38] and the relevance of the displayed item to their life using a pictorial representation of the item and the self [39]. The training is conducted twice a week (about 60 min per session) for six weeks. Through this implicit priming combined with the individualization of reinforcing events, we expect a decrease in compulsiveness in smoking behavior. The task will be conducted via an HTML-/JavaScript-based app which will be run by the participants from home.

### Baseline assessment (T1)

After screening, determining in- and exclusion criteria, and group allocation, all participants take part in a baseline assessment (Baseline, T1), which includes a written fully informed consent and the possibility to ask questions and a diagnostic interview [35] to validate the diagnosis of severe TUD and determine any comorbid mental disorders. They further complete various questionnaires, psychological assessments and neuropsychological assessments. An overview and schedule of all measurements conducted in the study is provided in Table 1. This is followed by an fMRI examination of about 1 h, which includes three experiments (cue-reactivity task, reward devaluation task and N-back task) and resting-state fMRI.

**Table 1:** Measurements and Interventions

*Baseline information/Medical screening*	
Sociodemographic data Internistic, neurological examination Drugs/pregnancy rapid test Carbon monoxide measurement Structured Clinical Interview for DSM-5	
*Questionnaires*	
Questionnaire of Smoking Urges (QSU-G, Müller et al. 2001) Fagerstrøm Test of Nicotine Dependence (Heatherton, Kozlowski, Frecker, & Fagerstrom, 1991) Craving Automated Scale for Cigarette (CAS-CS; adapted from CAS-A, Sabine Vollstädt-Klein, Leménager, Jorde, Kiefer, & Nakovics, 2015) Smoking Consequences Questionnaire (SCQ, Copeland, Brandon, & Quinn, 1995) Wisconsin Smoking Withdrawal Scale (WSWS, Welsch et al., 1999) Self-Report Habit Index (SRHI, Verplanken & Orbell, 2003) Center for Epidemiological Studies Depression Scale (CES-D/ADS, Hautzinger, Bailer, Hofmeister, & Keller, 2012) State-Trait-Anxiety Inventory (STAI, Laux, Glanzmann, Schaffner & Spielberger, 1981) Perceived Stress Scale (PSS, Cohen, Kamarck, & Mermelstein, 1983) BIS/BAS (Strobel et al. 2001) PANAS Trait / State (Watson & Clark, 1988) Barratt Impulsiveness Scale (BIS, Meule, Vögele, & Kübler, 2011) Expectation of therapy Goal Attainment scaling (Kiresuk, 1982)	
*Outcome consumption*	
Form 90 interview (Scheurich et al., 2005) Time to relapse number and percentage abstinent days CO-Test Cotinine hair analysis	
*Neuropsychological Assessment*	
Cambridge Gambling Task (CGT) Cambridge Automated Neuropsychological Test Automated Battery (CANTAB, Robbins et al., 1994) One Touch Stockings of Cambridge (OTS) Cambridge Automated Neuropsychological Test Automated Battery (CANTAB, Robbins et al., 1994) Intra-Extra Dimensional Set Shift (IED) Cambridge Automated Neuropsychological Test Automated Battery (CANTAB, Robbins et al., 1994) Spatial Working Memory (SWM) Cambridge Automated Neuropsychological Test Automated Battery (CANTAB, Robbins et al., 1994) Delay Reward Discounting Task (Koffarnus & Bickel (2014)) Dimensional Card Sorting Task (Zelazo et al. (2014)) Stop-Signal Task (Logan 1994) Dot-probe (Vollstädt-Klein et al. 2011) mit Nikotin-Stimuli Impliziter Assoziationstest (IAT, Greenwald, McGhee, & Schwartz, 1998) Kirby Delay Discounting Task (Kirby, 2009)	
*Functional magnetic resonance imaging*	
Structural MRT Resting state Cue reactivity (modifiziert nach S. Vollstädt-Klein, Kobiella, et al., 2011) Reward devaluation (Modifiziert nach Hogarth & Chase 2011) N-Back Task (Charlet et al. 2014	
*Interventions*	
Standard smoking cessation program (SCP) standard SCP plus Cognitive remediation treatment standard SCP plus implicit computer-based habit-modifying training	

*Cue reactivity task* To measure smoking-related cue reactivity, we will use a picture perception paradigm employing a block design similar to Vollstädt-Klein, Kobiella [40]. Smoking-related and neutral pictures will be taken from a validated picture series including both smoking cues with and without context. For smoking-related stimuli, different aspects of a smoking situations are depicted by presenting different external and internal context categories (e.g., people smoking in a pub vs. people smoking in a neutral environment). After each block, participants rate the intensity of their nicotine craving on a visual analogue scale. Re-test reliability for cue reactivity paradigms was shown to be good (with intraclass correlation coefficients above 0.75; [41]).

*Reward devaluation* We will examine the balance between goal-directed and habitual behaviour using a concurrent choice task for tobacco and chocolate points with a subsequent reward devaluation procedure, followed by a PIT phase with smoking pictures assumed to be conditioned stimuli in smokers ([42], established for human tobacco-seeking). Before the fMRI measurement, the participants take part in a concurrent choice training outside the scanner. Here, all individuals have the opportunity to win either cigarettes or chocolate bars by choosing one of two keys (counterbalanced by 50% chance within the subject). After each trial, the participants receive feedback on whether they have won a cigarette, chocolate bar or if nothing was won. Afterwards, a devaluation treatment takes place where participants are confronted with statements regarding the adverse consequences of smoking, which they have to evaluate from “not at all unpleasant” to “extremely unpleasant”. Then an extinction test is carried out immediately inside the fMRI scanner, which is similar to the concurrent choice task but without feedback after the key decision. Effects of devaluation are measured regarding alternative choice behaviour in the extinction phase. This phase is followed by a transfer phase, where cigarettes and chocolate can be won. However, sometimes the trials start with the presentation of visual cues including cigarettes or chocolate. This phase refers to a PIT paradigm because it can be assumed that the presented cues are conditioned cues (conditioned stimulus, CS) and impact on the unrelated instrumental task. The fMRI part of the procedure (i.e., extinction and PIT) has a duration of 22 min and has demonstrated high reliability (measured by split-half reliability, [43]) in AUD.

*N-back task* To assess working memory capacity as a domain of executive functioning, a classical N-back task will be performed in a block design [44]. Participants are asked to respond to previously displayed stimuli (numbers 1–4). In the 0-back control condition, subjects have to press the corresponding button of the currently displayed number, whereas the 2-back condition requires the recollection of a stimulus seen two stimuli before. Re-test reliability of this task was shown to be very good (intraclass correlation coefficient of 0.90; [45]).

*Resting-state* Participants will undergo an 8-min resting state fMRI examination while a fixation cross is shown and the participants are told to look at it to prevent falling asleep. Resting-state data can be used as predictors of drug-related responses [46].

*fMRI parameters* Scanning will be performed with a 3 T whole-body tomograph (MAGNETOM Prisma; Siemens, Erlangen, Germany). T2* weighted multi-band echo-planar images (mb-EPI) using a multi-band acceleration factor 6 will be acquired in a transversal orientation 20° clockwise to AC-PC-line covering the whole brain with the following parameters: TR = 869 ms, TE = 38 ms, 60 slices, slice thickness = 2.4 mm, voxel size 2.4 × 2.4 × 2.4 mm, no inter-slice gap, field of view (FoV) = 210 mm, matrix size 88 × 88, acquisition orientation T > C, interleaved slice order, acceleration factor slice = 6, flip angle = 58°, bandwidth = 1832 Hz/Px, prescan normalize, weak raw data filter, LeakBlock kernel, fat sat). Scanner sequences are provided by the Center for Magnetic Resonance Research (CMRR), University of Minnesota, Minneapolis, MN, USA (https://www.cmrr.umn.edu/multiband/). In addition, a T1-weighted 3D Magnetization Prepared—RApid Gradient Echo dataset consisting of 208 sagittal slices (slice thickness 1 mm, 1 × 1 × 1 mm voxel size, FOV 256 × 256 mm^2^, TR = 2000 ms, TE = 2.01 ms, TI = 800 ms, flip angle = 8°) will be acquired.

#### Neuropsychological assessments

Several neuropsychological assessments will be conducted via a self-written (by YS) HTML-/JavaScript-based app. The Implicit Association Task [47] measures the association bias. Participants sort smoking-related images, images of furniture, approach words, and avoidance words using a left and right key. Smoking approach associations are measured as the extent to which participants respond more quickly in trials in which smoking-related images are paired with approach words. The Kirby Delay Discounting Task [48] measures the delay discounting rate, which reflects the degree to which the value of an outcome decreases in the future. A lower discounting rate means that a person places a greater value on future consequences, which is associated with higher self-control. The task contains 27 trials, and in each trial the participant makes a choice between a smaller, immediate amount and a larger, delayed amount. The Iowa Gambling Task [49] measures decision-making, which is related to the processing of rewards. Participants are presented with four decks of cards. They can win or lose game money by selecting cards one at a time and learn over time that different decks entail different levels of risk and reward.

#### Questionnaires

Please see Table 1 for a description of the questionnaires applied throughout the study.

### Second investigation day (T2)

The second investigation day (T2) takes place after the six-week intervention period. Parallel to T1, participants will complete several fMRI tasks, neuropsychological assessments, and questionnaires. An overview and schedule of all measurements conducted in the study is provided in Table 1.

### Follow-up (T3)

Three months after the second investigation day, participants will be invited to a follow-up (T3). The same assessment as for the second investigation day will be administered, excluding fMRI. Cotinine, an established marker of tobacco smoke absorption, will be assessed from hair samples to assess nicotine intake during the last weeks. If the participants are not able or not willing to conduct the visit in person, a telephone follow-up will be suggested. This will allow to record instances of relapse and rates of nicotine consumption, but without neuropsychological testing. A detailed overview of the information collected at the specific time points is shown in Additional file 1.

### Statistical analyses

Overall, we aim to examine the effect of different training conditions on the imbalance between habitual and goal-directed behavior in individuals with TUD. Thus, different aspects of this question (e.g., reward devaluation, PIT-effect, cue-reactivity, neuropsychological functioning, and smoking behavior) will be analyzed and results will be integrated on a conceptual level.

Primary outcome measures include (1) change in the imbalance between goal-directed and habitual behavior using the reward devaluation fMRI paradigm; (2) change in implicit smoking-related associations using an implicit association task; (3) change in attentional bias to smoking using a dot-probe task; (4) change in smoking urges using the questionnaire of smoking urges; (5) change in working memory capacity using a spatial working memory task; (6) change in planning ability using the One Touch stockings of Cambridge task; (7) change in cognitive flexibility using the Internal–External Set Shifting task. Secondary outcome measures include (1) nicotine consumption level; (2) neural PIT effect; (3) neural cure-reactivity.

Pre-processing and statistical analyses of brain imaging data will be performed using SPM12 (Wellcome Centre for Human Neuroimaging at University College London, London, UK). After pre-processing, including motion correction, normalization to the Montreal Neurological Institute (MNI) template, and a spatial smoothing with Gaussian kernel of 8 mm full width at half maximum (FWHM), the images will be analyzed using the general linear model (GLM) approach at the first (single subject) and second (group) level. Regarding the reward devaluation fMRI task, the first level contrasts “cigarette versus chocolate” for the extinction phase and “cigarette versus chocolate versus no cue” for the PIT phase will be used for subsequent group analyses. The contrast “2-back versus 0-back” will be used for the 2-back fMRI task, and “tobacco versus neutral” (and also comparisons of the different smoking contexts) will be used for the cue reactivity fMRI task. To analyze the effect of the group (3 treatment conditions) and time-point (T1, T2), we will use a factorial 3 × 2 design on the behavioral and neural levels. Correction for multiple statistical testing will be done applying a family-wise error (FWE) correction at a level of *p* < 0.05. Further, t-tests and regression analyses will be conducted, e.g., to assess group differences at baseline that possibly need to be accounted for in subsequent analyses. Additional repeated measures analyses will be conducted to integrate data from T3 (follow-up) in order to assess the effect of group on abstinence and/or substance consumption.

In a second, exploratory and data-driven approach, we will use representational similarity analysis (RSA, [50]). With RSA, latent brain representations of psychological constructs can be examined not only across conditions and studies, but also between subgroups of individuals. In this study, we are using RSA to identify subgroups of smokers that might differ in biobehavioral activation patterns by exploring latent brain representations of goal-directed versus habitual behavior using above-described fMRI tasks.

## Discussion

Major detrimental health consequence of smoking and high relapse rates in individuals with TUD underline the still highly relevant need for further understanding of the disorder, i.e. neurobiological and behavioral mechanisms, and its treatment. With the present study project, we aim to contribute to this research gap by examining the effect of different therapeutic interventions on the imbalance of habitual and goal-directed behavior. We expect an imbalance between goal-directed and habitual behavior in treatment-seeking smokers, on the behavioral as well as on the neural level. Further a differential modification of this imbalance and its neural correlates is hypothesized when comparing SCP + CRT, SCP + ICHT and standard SCP. In addition, we expect a prediction of relapse by the imbalance between goal-directed and habitual behavior.

Of particular interest, not only for our study participants but the research community as well, we combine superiority testing of therapy modules with basic research on different levels (neurobiological, behavioral and psychological) and thus contribute to broadening the understanding of this complex disorder. As a major highlight, we postulate a positive effect of therapy add-ons on smoking behavior and abstinence rates due do adaptations und underlying neurobiological functioning. Further, we propose an additional, beneficial effect of our approach with regard to individualized medical interventions. If individual mechanisms of TUD prove to be identifiable, efficacious treatment options under study condition, e.g., cognitive versus habit training, might be administered more efficiently with respect to the individual’s needs—and hopefully prove to be equally effective in the naturalistic setting. Applying our proposed cognitive and computer-based habit training in groups further highlights the efficiency of these therapy add-ons. The repeated measures design can be considered a methodological strength as reduced error variance results from each participant being its own control [51].

Limitations of our study include the multitude of tasks and paradigms which might, on the one hand, complicate the integration of our findings into the overall conceptual topic of habitual and goal-directed behavior in TUD. On the other hand, these perspectives may equally represent an opportunity to broaden our knowledge of the complex disorder. Another limitation is the differences between interactive, real-life group therapy (SCP, SCP + CRT) and computer-based training (SCP + ICHT). The latter therapy add-on lacks well-known factors contributing to the positive outcome of group therapy, e.g., cohesiveness, catharsis, interpersonal learning, and universality of learning. However, adjusting the individual schedule in computer-based training might result in higher motivation to accomplish all training sessions.

## Supplementary Information

**Additional file 1: Figure S1**. SPIRIT Checklist for the schedule of enrolment, interventions, and assessments.

## Data Availability

For protection of personal rights, and due to the sensitivity of the clinical and neuroimaging data, data will not be made publicly available. The datasets used and analyzed during the current study are available from the corresponding author on reasonable request and in mutual agreements (e.g., regarding data protection and anonymization). Upon request, analysis procedures and codes will be shared with other researchers.

## Abbreviations

CRT: Cognitive remediation treatment
DSM: Diagnostic and statistical manual of mental disorders
DLPFC: Dorsolateral prefrontal cortex
EPI: Echo-planar images
FWE: Family wise error
FWHM: Full width at half maximum
fMRI: Functional magnetic resonance imaging
GLM: General linear model
ICHT: Implicit computer-based habit-modifying training
IFG: Inferior frontal gyrus
ICD: International classification of diseases
MNI: Montreal neurological Institute
PIT: Pavlovian-to-instrumental transfer
RSA: Representational similarity analysis
SCP: Smoking cessation program
TUD: Tobacco use disorder
