# Supplementary material for: Using computer-based habit versus chess-based cognitive remediation training as add-on therapy to modify the imbalance between habitual behavior and cognitive control in tobacco use disorder: protocol of a randomized controlled, fMRI study
Source: BMC Psychol. 2023 Jan 25;11:24. doi: 10.1186/s40359-023-01055-z (PMC9875438; doi:10.1186/s40359-023-01055-z)
Supplement: Supplementary file 1 — Additional file 1: Figure S1. SPIRIT Checklist for the schedule of enrolment, interventions, and assessments. [file 40359_2023_1055_MOESM1_ESM.doc]

Supplementary figure 1. SPIRIT Checklist for the schedule of enrolment, interventions, and assessments.*

|  | **Enrolment** | **Allocation** | **Post-allocation** | | |
| --- | --- | --- | --- | --- | --- |
| **TIMEPOINT** | ***-t_1_*** | **0** | ***t_1_*** | ***t_2_*** | ***t_3_*** |
| **ENROLMENT:** |  |  |  |  |  |
| **Eligibility screen** | X |  |  |  |  |
| **Informed consent** | X |  |  |  |  |
| **Allocation** |  | X |  |  |  |
| **ASSESSMENTS:** |  |  |  |  |  |
| **Baseline information/ Medical screening:** |  |  |  |  |  |
| *Sociodemographic data* |  |  | X |  |  |
| *Internistic, neurological examination* |  |  | X | X | X |
| *Drugs/* *pregnancy rapid test* |  |  | X | X | X |
| *Carbon monoxide measurement* |  |  | X | X | X |
| *Structured Clinical Interview for DSM-5* |  |  | X |  |  |
| **Questionnaires** |  |  |  |  |  |
| *Questionnaire of Smoking Urges (QSU-G, Müller et al. 2001)* |  |  | X | X | X |
| *Fagerstrøm Test of Nicotine Dependence (Heatherton, Kozlowski, Frecker, & Fagerstrom, 1991)* |  |  | X | X | X |
| *Craving Automated Scale for Cigarette (CAS-CS; adapted from CAS-A, Sabine Vollstädt-Klein, Leménager, Jorde, Kiefer, & Nakovics, 2015)* |  |  | X | X | X |
| *Smoking Consequences Questionnaire (SCQ, Copeland, Brandon, & Quinn, 1995)* |  |  | X | X | X |
| *Wisconsin Smoking Withdrawal Scale (WSWS, Welsch et al., 1999)* |  |  | X | X | X |
| *Self-Report Habit Index (SRHI, Verplanken & Orbell, 2003)* |  |  | X | X | X |
| *Center for Epidemiological Studies Depression Scale (CES-D/ADS, Hautzinger, Bailer, Hofmeister, & Keller, 2012)* |  |  | X | X | X |
| *State-Trait-Anxiety Inventory (STAI, Laux, Glanzmann, Schaffner & Spielberger, 1981)* |  |  | X | X | X |
| *Perceived Stress Scale (PSS, Cohen, Kamarck, & Mermelstein, 1983)* |  |  | X | X | X |
| *BIS/BAS (Strobel et al. 2001)* |  |  | X | X | X |
| *PANAS Trait / State (Watson & Clark, 1988)* |  |  | X | X | X |
| *Barratt Impulsiveness Scale (BIS, Meule, Vögele, & Kübler, 2011)* |  |  | X | X | X |
| *Expectation of therapy* |  |  | X | X | X |
| *Goal Attainment scaling (Kiresuk, 1982)* |  |  | X | X |  |
| **Outcome consumption** |  |  |  |  |  |
| *Form 90 interview (Scheurich et al., 2005)* |  |  | X | X | X |
| *Time to relapse* |  |  |  | X | X |
| *number and percentage abstinent days* |  |  | X | X | X |
| *CO-Test* |  |  | X | X | X |
| *Cotinine hair analysis* |  |  |  |  | X |
| **Neuropsychological tests** |  |  |  |  |  |
| *Cambridge Gambling Task (CGT) Cambridge Automated Neuropsychological Test Automated Battery (CANTAB, Robbins et al., 1994)* |  |  | X | X | X |
| *One Touch Stockings of Cambridge (OTS) Cambridge Automated Neuropsychological Test Automated Battery (CANTAB, Robbins et al., 1994)* |  |  | X | X | X |
| *Intra-Extra Dimensional Set Shift (IED) Cambridge Automated Neuropsychological Test Automated Battery (CANTAB, Robbins et al., 1994)* |  |  | X | X | X |
| *Spatial Working Memory (SWM) Cambridge Automated Neuropsychological Test Automated Battery (CANTAB, Robbins et al., 1994)* |  |  | X | X | X |
| *Delay Reward Discounting Task (Koffarnus & Bickel (2014) )* |  |  | X | X | X |
| *Dimensional Card Sorting Task (Zelazo et al. (2014)  )* |  |  | X | X | X |
| *Stop-Signal Task (Logan 1994)* |  |  | X | X | X |
| *Dot-probe (Vollstädt-Klein et al. 2011) mit Nikotin-Stimuli* |  |  | X | X | X |
| *Impliziter Assoziationstest (IAT, Greenwald, McGhee, & Schwartz, 1998)* |  |  | X | X | X |
| *Kirby Delay Discounting Task (Kirby, 2009)* |  |  | X | X | X |
| **Functional magnetic resonance imaging** |  |  |  |  |  |
| *Structural MRT* |  |  | X | X | X |
| *Resting state* |  |  | X | X | X |
| *Cue reactivity (modifiziert nach S. Vollstädt-Klein, Kobiella, et al., 2011)* |  |  | X | X | X |
| *Reward devaluation (Modifiziert nach Hogarth & Chase 2011)* |  |  | X | X | X |
| *N-Back Task (Charlet et al. 2014* |  |  | X | X | X |
| **INTERVENTIONS:** |  |  |  |  |  |
| *Standard smoking cessation program (SCP)* |  |  |  |  |  |
| *standard SCP plus Cognitive remediation treatment* |  |  |  |  |  |
| *standard SCP plus implicit computer-based habit-modifying training* |  |  |  |  |  |
